# A Mechanism of Prion Propagation

**DOI:** 10.1371/journal.pbio.0020360

**Published:** 2004-09-21

The key to any protein's function is its structure. Proteins first emerge as a linear strip of amino acids from the cellular protein-manufacturing machinery, and it is this primary sequence that determines a protein's ultimate conformation. Improperly folded proteins—which can gum up cells and, when secreted, tissues—are normally destroyed. But in a wide range of diseases, including prion (from proteinaceous and infectious) diseases and neurodegenerative diseases like Parkinson disease and Alzheimer disease, amyloid fibrils, or plaques—misshapen proteins that aggregate into characteristic rope-like configurations—accumulate in tissue.[Fig pbio-0020360-g001]

**Figure pbio-0020360-g001:**
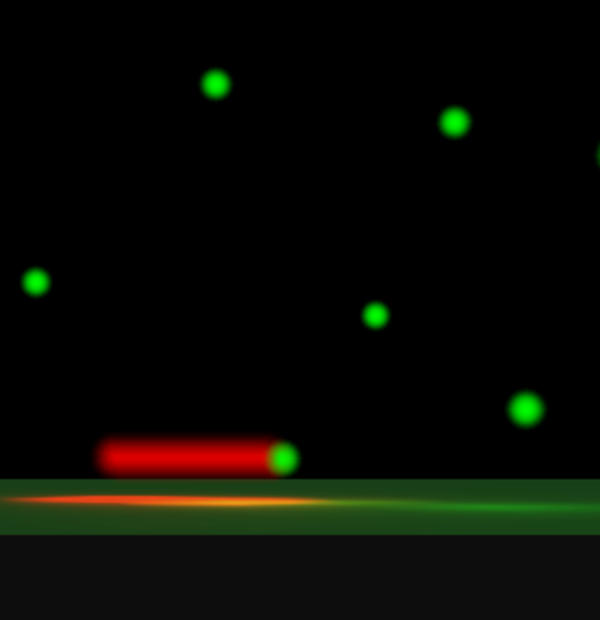
Schematic of single-molecule fluorescence experiment used to establish amyloids growth mechanism

When amyloid precursors and prions (pronounced PREE-ons) lose their normal conformation, they acquire the ability to infect their neighbors. Like molecular dominoes, the fall of one malformed protein precipitates the downfall of its neighbors, as one protein after another assumes the misshapen form of the first. Any chance of developing methods to contain the expansionist tendencies of these proteins depends on understanding the mechanism of propagation, an area of active research.

An abundance of small protein aggregates, called oligomers, is associated with amyloid fiber growth and formation. (Single proteins are called monomers; they “polymerize” into longer chains.) Mounting evidence suggests these so-called amyloid intermediates are the “toxic species” underlying amyloid diseases. The steps in amyloid formation, however, are unclear: Must amyloids follow a progression from monomer to oligomer to plaque? That is, are oligomers required for amyloid plaque formation? Using the yeast prion protein Sup35 to study how amyloids form, Jonathan Weissman and colleagues propose a model of amyloid plaque formation and show that it can indeed occur in the absence of the putative toxic oligomers.

In yeast, the Sup35 protein forms self-replicating aggregations reminiscent of amyloid formation and prion propagation. Though yeast aren't susceptible to prion diseases, they do assume what scientists call the yeast prion state. Two protein domains called NM together form self-propagating amyloid fibers that give rise to the yeast prion state. Oligomers, which are typically seen when other proteins form amyloids, have also been seen during this process, some of them near NM fiber ends. Weissman's team wanted to know what these oligomers were doing.

To investigate the role of oligomers in NM amyloid formation and growth, the researchers explored the relationship between monomer concentration and polymerization progress. Initially, fiber growth rate was tied to the concentration of NM monomers; but as concentrations increased, growth rate was moderated by NM conformational changes caused after binding to the fiber ends. Shaking the samples increased polymerization rate.

During polymerization reactions, the authors observed a pronounced pause, followed by an abrupt increase in polymerization rate. Since the length of the pause showed only a weak dependence on the concentration of monomers, Weissman and colleagues explain, this finding could not be explained by a simple model of nucleation polymerization, in which growth occurs monomer by monomer, emerging from a monomer “nucleus.”

Instead, Weissman and colleagues' findings support a model in which nucleated monomers initially support fiber growth, fibers undergo fragmentation, and monomers rapidly grow from the broken ends. Weissman and colleagues confirmed that the fibers were growing monomer by monomer by attaching to fragmented fiber ends with fluorescent microscopy, which can detect single molecules.

Though the authors do not rule out the possibility that oligomers could attach to the fiber ends as well, their results show that amyloid growth can occur independently of oligomers. Since many of the properties observed in Sup35 polymerization are evident in other amyloid-forming proteins, the model presented here may be shared as well. Future studies will have to explore this question, along with the issues of how oligomers figure into the process and how they cause disease. Weissman and colleagues raise the possibility that creating conditions that favor fiber growth while inhibiting oligomer formation might limit the toxic effects of amyloid plaques. The approaches outlined here should lay the foundation for exploring these questions in higher organisms.

